# Parasite‐mediated selection on host phenology

**DOI:** 10.1002/ece3.10107

**Published:** 2023-05-20

**Authors:** Hannelore MacDonald, Dustin Brisson

**Affiliations:** ^1^ Department of Biology University of Pennsylvania Philadelphia Pennsylvania USA

**Keywords:** adaptation, parasite, phenology

## Abstract

The timing of seasonal activity, or phenology, is an adaptive trait that maximizes individual fitness by timing key life events to coincide with favorable abiotic factors and biotic interactions. Studies on the biotic interactions that determine optimal phenology have focused on temporal overlaps among positively‐interacting species such as mutualisms. Less well understood is the extent that negative interactions such as parasitism impact the evolution of host phenology. Here, we present a mathematical model demonstrating the evolution of host phenological patterns in response to sterilizing parasites. Environments with parasites favor hosts with shortened activity periods or greater distributions in emergence timing, both of which reduce the temporal overlap between hosts and parasites and thus reduce infection risk. Although host populations with these altered phenological patterns are less likely to mature and reproduce, the fitness advantage of parasite avoidance can be greater than the cost of reduced reproduction. These results illustrate the impact of parasitism on the evolution of host phenology and suggest that shifts in host phenology could serve as a strategy to mitigate the risk of infection.

## Graphical abstract

Environments with parasites favor hosts with shifted phenology to reduce the temporal overlap between hosts and parasites and thus reduce infection risk. Although host populations with altered phenological patterns are less likely to mature and reproduce, the fitness advantage of parasite avoidance can be greater than the cost of reduced reproduction. These results illustrate the impact of parasitism on the evolution of host phenology and suggest that shifts in host phenology could serve as a strategy to mitigate the risk of infection.

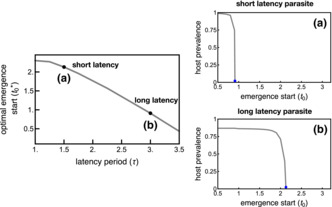

## 1 INTRODUCTION

The timing of seasonal activity, or phenology, is an adaptive trait that maximizes individual fitness by timing key life events within a season. The phenological pattern that maximizes individual fitness is impacted by many abiotic environmental factors that determine when activities such as breeding, foraging, and migration are favorable (Forrest & Miller‐Rushing, 2010). Similarly, biotic factors such as inter‐species interactions impact phenology by selecting seasonal activity patterns that overlap temporally with food sources and mutualists (e.g., plants and pollinators) (Elzinga et al., 2007; Kochmer & Handel, 1986; Sargent & Ackerly, 2008). The multitude of seasonally varying abiotic and biotic factors preclude most species from optimizing their phenological pattern to each factor simultaneously (Pau et al., 2011; Van Schaik et al., 1993). For example, plants must balance the costs of herbivory with the benefits of pollination as the seasonal activity of insect herbivores and pollinators often overlap (Van Schaik et al., 1993). Although many abiotic and biotic factors that impact phenology have been investigated, the impacts of parasitism remain relatively under‐explored.

Parasites are an important driver of the evolution of many phenotypes. For example, nearly all species have evolved energetically costly defenses against infection including innate and adaptive immune responses (Rimer et al., 2014). Parasite‐induced shifts in host phenology could serve as an alternative defense against infection, although it is unclear whether the benefits associated with reducing infection risks caused by phenological shifts would surpass the opportunity costs incurred due to missing other seasonal events (e.g., reproduction and development time) (Alexander et al., 1993; Biere & Antonovics, 1996). Nevertheless, there are several examples in which certain host phenological patterns are correlated with a decrease in infection risk. For example, late‐flowering *Silene alba* have lower infection rates with the sterilizing anther‐smut fungus (*Ustilago violacea*) than early‐flowering plants (Alexander, 1989, 1990; Biere & Antonovics, 1996; Biere & Honders, 1996). Late‐flowering plants do, however, have lower flower production, a measure of plant reproductive fitness (Biere & Antonovics, 1996; Biere & Honders, 1996).

Changes in host phenology could reduce infection rates in multiple ways. For example, host activity patterns that do not temporally overlap with the peak in parasite abundance directly decrease infection risk. This mechanism is supported empirically in several systems. Namely, temporal coordination between hosts and parasites is a major determinant of trematode infections of the amphibian *Pseudacris regilla*, parasitic wasp infections in apple maggot flies, and nematode infections in sheep (Feder, 1995; Gethings et al., 2015; McDevitt‐Galles et al., 2020). Host phenological patterns could also be suboptimal for parasite fitness resulting in ecological feedback that reduces host infection risks in future years. Although host phenological patterns likely impact infection risk, few studies have focused on how host phenological patterns could have arisen through selection to decrease infection risk.

We develop a mathematical model to assess the impact of parasitism on the evolution of host phenology. Specifically, the model evaluates the fitness advantages of avoiding infection through alternate host activity patterns along with the potential fitness costs incurred. In this model, the fitness of hosts emerging later is lower due to reduced reproductive potential, but these hosts also have lower risks of sterilizing infections. Given the assumptions of the model, late‐shifted host phenological patterns can be an adaptive strategy resulting in greater individual fitness and population densities in environments with parasites. That is, the presence of parasites can select for late‐shifted host phenological patterns and an increased duration in the host emergence period even though both phenological changes reduce the reproductive potential of individual hosts. This study demonstrates that parasitism can impact the evolution of host phenology and provides a framework for predicting the impact of future changes in parasite abundance on host species.

## 2 MODEL DESCRIPTION

**FIGURE 1:**
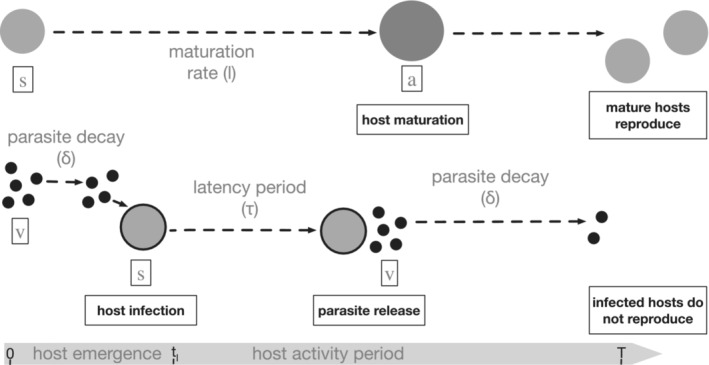
Diagrammatic representation of host infection and maturation within each season. Juvenile hosts (s) emerge at a constant rate between time $t=t_{0}$ and $t=t_{0}+t_{l}$ and develop into adult hosts (a) at rate l. Only hosts that have matured by the end of the season contribute progeny that will emerge in the next season. All parasites (v) emerge at the beginning of the season $\left(t=0\right).$ The rate of infection is density‐dependent such that the majority of the first round of infections occur near the beginning of the season when susceptible host and free parasite densities are high. New parasites are released at time τ postinfection. If τ is short enough, more than one generation of infections can occur within the season. Parasite progeny that survive in the environment to the end of the season comprise the parasite population that emerges in the following season.

The model describes the transmission dynamics of a free‐living, sterilizing parasite that infects seasonally available juvenile hosts (Figure 1). The susceptible host cohort, $\hat{s}\left(n\right)$, enters the system as juveniles at the beginning of season n. The timing of juvenile host emergence during the season, that is, host phenology, is given by the function $g\left(t_{0},t_{l}\right)$, which describes when host emergence begins (at $t=t_{0}$) and the length of time over which $\hat{s}\left(n\right)$ emerge ($t_{l}$). Hosts have nonoverlapping generations and are alive for one season. The parasite, $v_{n}$, infects juvenile hosts, $s_{n}$, who are susceptible to infection, analogous to, for example, baculoviruses of forest *Lepidoptera* (Baltensweiler et al., 1977; Bilimoria, 1991; Dwyer, 1994; Dwyer & Elkinton, 1993; Woods & Elkinton, 1987) and univoltine insects parasitized by ichneumonids (Campbell, 1975; Delucchi, 1982; Kenis & Hilszczanski, 2007). The parasite releases new infectious progeny only after a set latency period (τ), which determines the number of rounds of infection the parasite completes within a season. The parasite completes one round of infection per season if the parasite has a long latency period (long τ) while the parasite completes multiple rounds of infection per season if the parasite has a short latency period (short τ). Juvenile hosts mature into the adult developmental stage, $a_{n}$, that is resistant to infection during the season. Adult hosts reproduce in between‐seasons to give rise to next season's host cohort ($\hat{s}\left(n+1\right)$). We assume infected $s_{n}$ hosts cannot reproduce and thus ignore their progression to the reproductively active $a_{n}$ stage.

The duration of each season extends from t=0 to t=T. Time units are not specified in order to maintain the generality of the model across disease systems. It is expected that the relevant time unit will be in months for many disease systems, corresponding to spring and summer (Baltensweiler et al., 1977; Danks, 2006; Donovan, 1991; Grant & Shepard, 1984; Takasuka & Tanaka, 2013) and weeks for other disease systems (Cummins et al., 2011; Dalen, 2013; Danks, 2006). The initial conditions in the beginning of the season are $s_{n}\left(0\right)=0,v_{n}\left(0\right)=\hat{v}\left(n\right)=\gamma v_{n-1}\left(T\right)$ where $\hat{v}\left(n\right)$ is the size of the starting parasite population introduced at the beginning of season n, which is the product of the number of parasites progeny remaining at the end of season (t=T) in season n−1 and the probability that those parasites survive between‐seasons (γ). The transmission dynamics in season n are given by the following system of delay differential equations (all parameters are described in Table 1):

(1a) $$\frac{{\mathrm{ds}}_{n}}{\mathrm{dt}}=\hat{s}\left(n\right)g\left(t_{0},t_{l}\right)-\mu_{s}s_{n}\left(t\right)-\alpha s_{n}\left(t\right)v_{n}\left(t\right)-{\mathrm{ls}}_{n}\left(t\right),$$

(1b) $$\frac{{\mathrm{da}}_{n}}{\mathrm{dt}}={\mathrm{ls}}_{n}\left(t\right)-\mu_{a}a_{n}\left(t\right),$$

(1c) $$\frac{{\mathrm{dv}}_{n}}{\mathrm{dt}}=\alpha \beta e^{-\mu_{s}\tau }s_{n}\left(t-\tau \right)v_{n}\left(t-\tau \right)-\delta v_{n}\left(t\right),$$

where μ is the host death rate, l is the host maturation rate, δ is the decay rate of parasites in the environment, α is the density‐dependent transmission rate, and τ is the delay between host infection and host death. The term $e^{-\mu_{s}\tau }$ is the proportion of infected hosts who survive the latency period. β is the number of parasites released upon host death at the end of the latency period (τ). We make the common assumption for free‐living parasites that the removal of parasites through transmission (α) is negligible (Anderson & May, 1981; Caraco & Wang, 2008; Dwyer, 1994), that is, (1c) ignores the term $-\alpha s_{n}\left(t\right)v_{n}\left(t\right)$.

**TABLE 1:** Model parameters and their respective values.

Parameter	Description	Value
s	Susceptible, juvenile hosts	State variable
a	Adult hosts	State variable
v	Parasites	State variable
$\hat{v}\left(n\right)$	Starting parasite population in season n	State variable
$\hat{s}\left(n\right)$	Host cohort in season n	State variable
$t_{0}$	Start of host emergence	Time (evolves), 0 when held constant
$t_{l}$	Length of host emergence period	Time (evolves), 0.5 when held constant
T	Season length	4
n	Season number	Varies
α	Transmission rate	$10^{-6}$/(parasite × time)
β	Number of parasites produced upon host death	400 parasites
δ	Parasite decay rate in the environment	2 parasites/parasite/time
$\mu_{s}$	Juvenile host death rate	0.2 hosts/host/time
$\mu_{a}$	Adult host death rate	0.015 hosts/host/time
l	Host maturation rate	1 hosts/host/time
τ	Latency period (1/virulence)	Time, varies from 1 to 3.5
γ	Parasite between‐season survival probability	0.9
ε	Host between‐season survival probability	0.85
σ	Host fecundity	600 hosts
ρ	Density‐dependent parameter	0.0001

The function $g\left(t_{0},t_{l}\right)$ is a probability density function that captures the per‐capita host emergence rate by specifying the timing and length of host emergence. We use a uniform distribution ($U\left(•\right)$) for simplicity, although other distributions are expected to have qualitatively similar results (MacDonald et al., 2022).

(2) $$g\left(t_{0},t_{l}\right)=\left(\begin{array}{ll} 0 & t_{0}<t\\ \frac{1}{t_{l}} & t_{0}\leq t\leq t_{0}+t_{l}\\ 0 & t_{0}+t_{l}<t\leq T \end{array}\right.$$

$t_{0}$ denotes the start of first host emergence, $t_{l}$ denotes the length of the host emergence period, and T denotes the season length. The host cohort emerges from $t_{0}\leq t\leq t_{0}+t_{l}.$ $\hat{s}\left(n\right)$ is a function of the density of mature hosts that survive to the end of the previous season ($a_{n-1}\left(T\right)$), given by

(3) $$\hat{s}\left(n\right)=\frac{\epsilon \sigma a_{n-1}\left(T\right)}{1+\rho a_{n-1}\left(T\right)},$$

where ϵ is the probability that hosts survive to the next season, σ is host reproduction, and ρ is the density‐dependent parameter.

Infections that have not completed the full latency period (τ) by the end of the season do not release progeny. Background mortality arises from predation or some other natural cause. We assume that infected hosts that die from background mortality do not release parasites because the parasites are either consumed or the latency period corresponds to the time necessary to develop viable progeny (Wang, 2006; White, 2011).

In previous work on a similar model, we derived an analytical expression for end‐of‐season host and parasite density ($a_{n}\left(T\right)$, $v_{n}\left(T\right)$, respectively) (MacDonald & Brisson, 2022a). However, we can only solve system (1) in the current framework analytically if τ is long enough that parasites only complete one infection generation per season. We outline the analysis for this case in Appendix S1A. All other results were found by performing numerical computations.

We have shown previously that host carryover can generate a feedback between parasite fitness and host demography that drives quasiperiodic dynamics for some parameter ranges (MacDonald & Brisson, 2022a, 2022b). In this study, we found no evidence that host–parasite cycling occurs for the parameter ranges considered, that is, the dynamics always eventually settle to stable end‐of‐season densities.

### 2.1 Host evolution

To study how host phenology traits adapt when challenged by parasites, we use evolutionary invasion analysis (Geritz et al., 1998; Metz et al., 1992). We first extend system (1) to follow the invasion dynamics of a rare mutant host

(4a) $$\frac{{\mathrm{ds}}_{n}}{\mathrm{dt}}=\hat{s}\left(n\right)g\left(t_{0},t_{l}\right)-\mu_{s}s_{n}\left(t\right)-\alpha s_{n}\left(t\right)v_{n}\left(t\right)-{\mathrm{ls}}_{n}\left(t\right),$$

(4b) $$\frac{{\mathrm{da}}_{n}}{\mathrm{dt}}={\mathrm{ls}}_{n}\left(t\right)-\mu_{a}a_{n}\left(t\right),$$

(4c) $$\frac{{\mathrm{ds}}_{n,m}}{\mathrm{dt}}={\hat{s}}_{m}\left(n\right)g\left(t_{0m},t_{\mathrm{lm}}\right)-\mu_{s}s_{n,m}\left(t\right)-\alpha s_{n,m}\left(t\right)v\left(t\right)-{\mathrm{ls}}_{n,m}\left(t\right),$$

(4d) $$\frac{{\mathrm{da}}_{n,m}}{\mathrm{dt}}={\mathrm{ls}}_{n,m}\left(t\right)-\mu_{a}a_{n,m}\left(t\right),$$

(4e) $$\frac{{\mathrm{dv}}_{n}}{\mathrm{dt}}=\alpha \beta e^{-\mu_{s}\tau }v_{n}\left(t-\tau \right)\left(s_{n}\left(t-\tau \right)+s_{n,m}\left(t-\tau \right)\right)-\delta v_{n}\left(t\right),$$

where m subscripts refer to the invading mutant host and its corresponding traits.

To estimate the invasion fitness of a single mutant, we define mutant invasion fitness as the density of $s_{n+1,m}$ emerging in season n+1 (${\hat{s}}_{m}\left(n+1\right)$) produced by a single invading mutant host (${\hat{s}}_{m}\left(n\right)=s_{n,m}\left(0\right)=1$) introduced in season n in the environment set by the resident host at equilibrium density ${\hat{s}}^{*}$. That is, mutant invasion fitness is equivalent to ${\hat{s}}_{m}\left(n+1\right)$ that were produced in season n by $s_{n,m}$ who successfully matured by the end of the season $a_{n,m}\left(T\right)$. Following the same approach as in previous analyses (MacDonald et al., 2022; MacDonald & Brisson, 2022a, 2022b), the mutant host invades in a given host phenological scenario if ${\hat{s}}_{m}\left(n+1\right)$ is greater than or equal to the initial ${\hat{s}}_{m}\left(n\right)=s_{n,m}\left(0\right)=1$ introduced at the start of season n $\left({\hat{s}}_{m}\left(n+1\right)\geq 1\right)$. Optimal phenological traits ($t_{0}^{*}$, $t_{l}^{*}$) are those that invade and replace populations with alternative trait values when rare and also prevent invasion, while at equilibrium, from mutants with different trait values. We are only able to solve the current model analytically when hosts are challenged by parasites that complete one round of infection per season (Appendix S1B). For consistency, we determine all host evolutionary endpoints numerically (Appendix S1C.)

The invasion analyses predict the evolution of the timing that host emergence begins ($t_{0}$) and the host emergence period duration ($t_{l}$) when hosts are in an environment with a parasite with a defined latency period length. Optimal trait values for $t_{0}$ and $t_{l}$ thus balance the cost of parasite infection with the cost of not reproducing. This study followed the evolution of $t_{0}$ or $t_{l}$ in isolation, rather than the simultaneous evolution of both traits. Parameter values are listed in Table 1.

## 3 RESULTS

**FIGURE 2:**
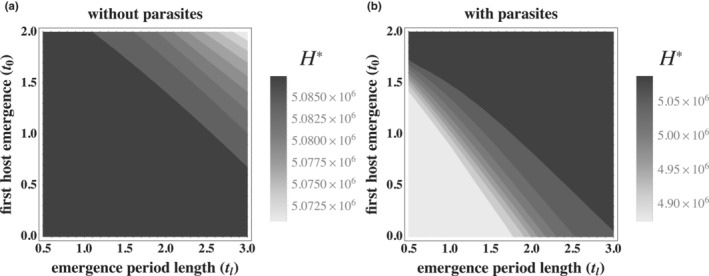
Host populations that emerge late during the season or have long emergence periods have relatively lower densities (lower reproductive fitness) in environments without parasites (a) but substantially greater densities in environments with parasites (b). Contour plots depict equilibrium host density (${\hat{s}}^{*}$, calculated using equation 3) for combinations of the timing at which hosts first emerge ($t_{0}$) and the length of the host emergence period ($t_{l}$). Note the when $t_{0}+t_{l}>T$, not all hosts emerge before the end of the season. τ=2, n=100, all other parameters are the same as in Table 1. Note that legends are at different scales.

Host emergence near the beginning of the season maximizes the probability of maturing and producing offspring (Figure 2). By contrast, later‐emerging hosts are less likely to reach reproductive maturity due to their shorter activity period. The costs associated with reducing the probability of reaching reproductive maturity, however, can be less than the costs associated with the risk of parasitic infection, which are greatest in early‐emerging hosts. That is, hosts that emerge early are at higher risk of infection from the sterilizing parasites that are most abundant at the beginning of the season and subsequently decay. Thus, the presence of parasites can create a selective pressure favoring later host emergence despite the cost of reducing the probability of reaching reproductive maturity.

**FIGURE 3:**
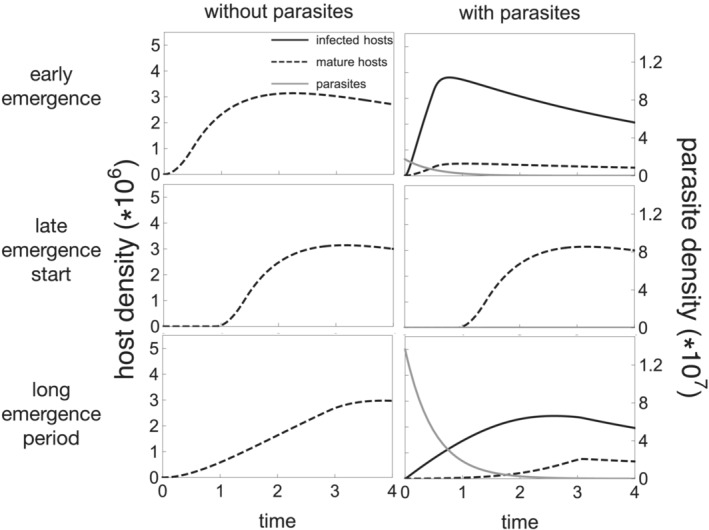
Late‐shifted phenological activity patterns maximize host density in environments with parasites. Top row: equilibrium host density (i.e., the size of the host cohort calculated using equation 3) is higher in environments without parasites (${\hat{s}}^{*}=5.08*10^{6}$) compared with environments with parasites (${\hat{s}}^{*}=4.94*10^{6}$) when $t_{0}=0,\;t_{l}=0.5$. Early phenology grants high host density in the absence of parasites but results in most hosts becoming infected in environments with parasites as parasite density is high early in the season. Middle row: host populations that begin emerging late in environments with parasites increase their density compared with populations that emerge at t=0 by avoiding high parasite densities early in the season and effectively eliminating infections (${\hat{s}}^{*}=5.08*10^{6}$, $t_{0}=0.91$). Host population density is the same regardless of whether parasites are introduced when $t_{0}=0.91$ as the host phenological pattern does not support parasites. Bottom row: host populations with longer emergence periods ($t_{l}=2.88$) also increase their density compared to populations with shorter emergence periods ($t_{l}=0.5$) as hosts that emerge late in the cohort avoid high early season parasite densities (${\hat{s}}^{*}=5.02*10^{6}$ when $t_{l}=2.88$). However, longer emergence periods do not drive parasites extinct, thus gains in host density do not fully recover losses in host density due to parasitism (${\hat{s}}^{*}=5.08*10^{6},\;t_{l}=2.88$ in environments without parasites.) Gray line: first generation of parasites in the season, black line: infected hosts, dashed black line: mature hosts. τ=3, n=150 (population dynamics reached stable end‐of‐season densities), all other parameters are the same as in Table 1.

**FIGURE 4:**
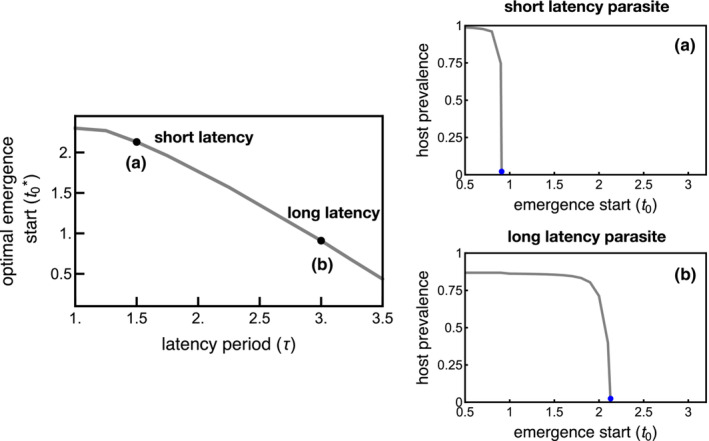
Late emergence starts are adaptive for host species in environments with parasites with short latency periods (short τ) by reducing host infection prevalence. (a) A later emergence start ($t_{0}^{*}=2.13$) is adaptive in environments with parasites that have short latency periods (τ=1.5) as infection risk is essentially eliminated (host infection prevalence is close to 0). (b) Only a slightly later emergence start ($t_{0}^{*}=0.91$) is necessary to eliminate infections from long latency period parasites (τ=3). $t_{0}$ is the time within the season that host emergence begins (i.e., emergence begins at $t=t_{0}$). Dots correspond to the optimal emergence start ($t_{0}^{*}$) when parasites have short latency (a) and long latency (b). n=300, in the absence of parasites, $t_{0}^{*}=0$, all other parameters are the same as in Table 1.

Shifts in the start of the activity period can increase host reproductive fitness through increased host density both directly and indirectly (Figure 3). Shifting the start of the activity period later in the season directly reduces the risk of infection for all members of the population as the risk of infection is greatest at the beginning of the season when parasite density is at its maximum. Late‐shifted activity periods have two indirect impacts on host density that both limit parasite reproductive success resulting in a smaller parasite population the following season. First, there are fewer infections in host populations with late‐shifted activity periods, which limit the number of parasite progeny produced. Second, parasite population sizes are limited as the infections that do occur are less likely to release progeny due to the set latency period between infection and progeny production. These impacts on host density due to later emergence timing occur with all tested parasite virulence phenotypes (τ), although the impacts are quantitatively different (Figure 4).

The optimal timing of first host emergence can result in the near‐elimination of infections (Figure 4). Evolutionary shifts toward even later timing of first emergence in host phenological environments with few parasite infections are not selectively favored as the risk of infection is already minimal. The optimal host emergence start time is a function of the length of the parasite latency period (Figure 4). For example, very late host emergence start times are necessary to limit host infections that produce progeny and dramatically diminish infections from parasites with short latency periods (Figure 4a). That is, large shifts toward later host emergence can cause infections to occur late enough in the season to prevent most short latency period parasites from releasing parasite progeny. Only small evolutionary shifts in the host emergence start time are necessary to limit infections from most parasites with long latency periods (Figure 4b).

**FIGURE 5:**
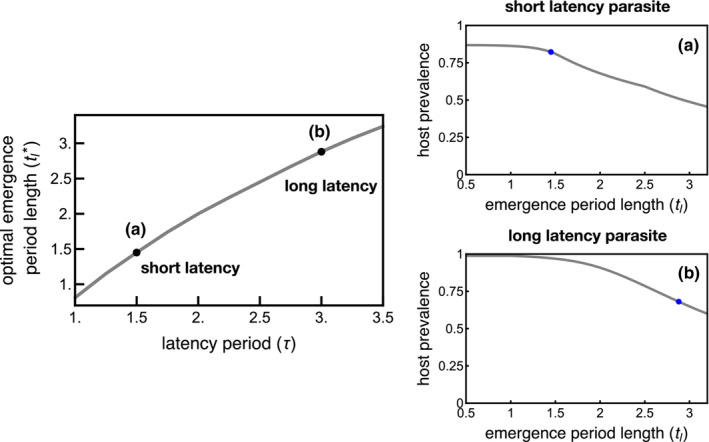
Long emergence period lengths are adaptive for host species in environments with parasites with long latency periods (long τ) but do not eliminate infection risk as measured by host infection prevalence. (a) A slightly longer emergence period length ($t_{l}^{*}=1.45$) is adaptive in environments with parasites that have short latency periods (τ=1.5). (b) A long emergence period ($t_{l}^{*}=2.88$) is adaptive in environments with parasites that have long latency periods (τ=3) to decrease the risk of infection. $t_{l}$ is the length of the host emergence period. Dots correspond to the optimal emergence period length ($t_{l}^{*}$) when parasites have short latency (a) and long latency (b). n=300, in the absence of parasites, $t_{l}^{*}=0.5$ (minimum possible emergence period length), all other parameters are the same as in Table 1.

Host populations with widely distributed emergence periods can also reduce the average risk of infection with long latency period parasites (Figure 5b). That is, the host individuals emerging later in the season have low infection risk such that host populations with larger proportions of hosts emerging later in the season have lower average infection rates overall. Further, infections that occur late in the season are less likely to result in parasite progeny, which limits the parasite population size the following season. Evolutionary shifts in host emergence periods, however, do not eliminate infection risk. The proportion of hosts emerging early regularly acquire infections that produce parasite progeny (Figure 3). Thus, long emergence periods are adaptive as a means to reduce host infection risk, but the impact is not as strong as shifting back the start time of the entire host cohort (i.e., late $t_{0}$). Nevertheless, host populations with greater proportions of later‐emerging individuals can have a selective advantage and invade systems with shorter emergence periods.

Intermediate host emergence period lengths optimize host evolutionary fitness in the presence of short latency period parasites (Figure 5a). Specifically, both early‐ and late‐emerging hosts are at a high infection risk when the parasite latency period is short enough that parasites can complete multiple rounds of infection within each season. Early‐emerging hosts risk infection by parasites produced the prior season while later‐emerging hosts risk infection by progeny released from infections of early‐emerging hosts. The late‐emerging hosts in populations with long emergence periods provide naïve hosts for the progeny produced from early infections, which can limit the benefit of even longer emergence period lengths.

## 4 DISCUSSION

The selection pressure created by parasites can be sufficiently strong to drive evolutionary shifts in host phenology (Figures 4 and 5). Evolution in the timing at which a host population begins to emerge (Figure 4), as well as the duration of the emergence period (Figure 5), can increase host fitness in some environments due to selection pressures generated by sterilizing parasites. In the absence of parasites, host populations that emerge early and have short emergence periods have the greatest possible fitness as all individual hosts have sufficient time to mature into adults and reproduce (Figure 2). However, the risk of infection is greatest for hosts that emerge early in environments with parasite populations, favoring the evolution of later‐emerging host populations.

Host populations that emerge late in the season can have increased host fitness immediately and over several generations. Infection risk is lower for later‐emerging hosts, as infectious parasite populations are largest at the beginning of the season, resulting in greater evolutionary fitness even when parasite populations are large (Figure 4). Additionally, environments with late‐emerging host populations can drive local parasite populations near extinction over successive generations (Figure 4). Thus, evolutionary changes in the timing of first emergence are an evolutionary mechanism that can efficiently reduce infection risk and increase host fitness. These results suggest that, for example, late‐flowering phenology in *Silene alba* could be an adaptation to avoid *Ustilago violacea* infection (Alexander, 1989; Biere & Antonovics, 1996; Biere & Honders, 1996). The evolutionarily optimal time at which hosts first emerge depends upon the latency period of the parasite, although reductions in parasite population sizes occur for all parasites regardless of their latency period. While these results do not yet have empirical support, they could be tested in systems such as *S. alba*‐*U. violacea* by measuring parasite densities in environments where host species have different phenological patterns.

The length of the host emergence period can also increase host evolutionary fitness by decreasing infection risk. However, long host emergence periods only effectively reduce the average infection risk of long latency period parasites by shifting some infections late enough in the season to prevent the release of new parasites. Further, evolutionary increases in host emergence period duration are a less‐effective means of decreasing infection risk than evolutionary changes in the timing of first emergence. Interestingly, long host emergence periods can also serve as a bet‐hedging strategy in response to temporally variable environments (Simons, 2014) such that the benefits of bet‐hedging could come at the cost of increased infection risk.

The host phenological traits under selection in this study (emergence start and period length) could represent several activity types that impact host contact with parasites in natural systems. For example, this model could apply to host species that undergo diapause during unfavorable environmental conditions, as in many disease systems with insect hosts (Delucchi, 1982; Donovan, 1991; Grant & Shepard, 1984; Takasuka & Tanaka, 2013). Similarly, host phenology could represent the start and length of time that host species are born (Campbell, 1975; Danks, 2006; Kenis & Hilszczanski, 2007). These results could also apply to disease systems with hosts that migrate to the same area each season, with emergence start and emergence period length representing the start and length of time over which hosts migrate into an area, respectively. Theory developed on the evolution of host migration has some parallels to the results presented herein that host–parasite intensity is predicted to select for partial host migration (Balstad et al., 2021; Shaw & Binning, 2016)—a prediction that is matched by empirical data on reindeer (Folstad et al., 1991) and fish migration (Poulin et al., 2012; Sikkel et al., 2017).

The presented model assumes that host phenology can evolve in response to parasite infection risks without considering parasite evolution. This strict assumption, which likely impacts long‐term dynamic and equilibrial results, is unlikely to occur in any natural system. For example, phenological evolution in apple maggot fly populations driven by parasitic wasps is followed by the phenological evolution of wasp species (Feder, 1995; Hood et al., 2015). Similarly, prior theoretical work has demonstrated that host phenological patterns impact the evolution of latency period phenotypes in parasites (MacDonald et al., 2022). Extending this model framework to permit the evolutionary change in both host phenology and parasite latency period could result in a chase‐away selection regime where the presence of parasites selects for later host phenological patterns that in turn selects for shorter latency periods, leading to selection for even later phenology. Alternatively, this model extension could result in a cycle of compensatory host and parasite selective sweeps in which shorter latency periods select for earlier host emergence leading to selection for longer parasite latency periods and subsequent selection for earlier phenological patterns.

The results of the present study could change if certain strict assumptions are relaxed or different parameter ranges are considered. For example, phenological avoidance of infections could cause hosts to miss crucial interactions with mutualists or increase their predation risk. Additionally, host populations that evolve earlier emergence—who would suffer an increased mortality rate—could coexist with host populations that emerge late—who suffer a reduced reproduction probability—analogous to prior reports on the evolution of species phenology in response to density‐dependent competition (Metcalf et al., 2015). Further, host emergence start and emergence period length could depend on one another, thus an extension that studied their simultaneous evolution could come to different conclusions. For example, earlier host emergence start times may be adaptive if this trait is correlated with emergence period length as the current study suggests that selection for long emergence periods is weak.

Similarly, parameter ranges that increase infections would likely select for stronger shifts in host phenology (e.g., larger numbers of parasites produced upon host death, lower parasite decay rates) while parameter ranges that decrease the density of susceptible hosts would likely select for weaker shifts in host phenology by decreasing the number of infections (e.g., increased host death and maturation rates.) We explore the impact of changing transmission rates (α), host fecundity $\left(\sigma \right)$, and the strength of the host density‐dependent parameter (ρ) in Appendix S1D. We find that the host density‐dependent parameter strongly impacts optimal host phenology while only extremely small transmission rates and extremely small host fecundity values select for smaller shifts in host phenology. Future work will consider these extensions in more detail to move toward the goal of developing a more complete theory on the role infection can play in host adaptation in seasonal environments.

Parasites are important drivers of host evolution through their impact on host fitness. While the majority of research on the impacts of parasites on hosts focuses on the evolution of immune defenses, the current study presents an alternative evolutionary mechanism in the form of altered seasonal patterns to reduce infection risk. The theory developed here predicts that parasitism can drive host phenology evolution, providing a framework to determine how future changes in parasite distribution and abundance could impact host evolution.

## AUTHOR CONTRIBUTIONS

**Hannelore MacDonald:** Conceptualization (lead); formal analysis (lead); investigation (lead); methodology (lead); writing – original draft (lead); writing – review and editing (equal). **Dustin Brisson:** Funding acquisition (lead); writing – original draft (supporting); writing – review and editing (equal).

## FUNDING INFORMATION

This work was supported by the National Institutes of Health (T32AI141393 (HM) R01AI142572 (DB), R01AI097137 (DB)); the National Science Foundation (DEB‐1354184 (DB)); and the Burroughs Wellcome Fund (1012376 (DB)).

## CONFLICT OF INTEREST STATEMENT

The authors declare no conflicts of interest.

### OPEN RESEARCH BADGES

This article has earned Open Data, Open Materials and Preregistered Research Design badges. Data, materials and the preregistered design and analysis plan are available at [https://github.com/hanneloremac/Parasite‐mediated‐selection‐on‐host‐phenology].

## PERMISSION TO REPRODUCE MATERIALS FROM OTHER SOURCES

None.

## Supporting information


Appendix S1.

## Data Availability

Code is available on the Github repository: https://github.com/hanneloremac/Parasite‐mediated‐selection‐on‐host‐phenology.
