# Supplementary material for: Parasite‐mediated selection on host phenology
Source: Ecol Evol. 2023 May 20;13(5):e10107. doi: 10.1002/ece3.10107 (PMC10199498; doi:10.1002/ece3.10107)
Supplement: Supplementary file 1 — Appendix S1. [file ECE3-13-e10107-s001.pdf]

Appendix A

In Appendix A we find analytical solutions for equations (1a-c) from the main text when parasites can only complete one round of infection per season due to a long latency period (τ).

$$\frac{ds_n}{dt} = \hat{s}(n)g(t_0, t_l) - \mu_s s_n(t) - \alpha s_n(t)v_n(t) - l s_n(t), \tag{A.1a}$$

$$\frac{da_n}{dt} = l s_n(t) - \mu_a a_n(t), \tag{A.1b}$$

$$\frac{dv_n}{dt} = \alpha\beta e^{-\mu_s \tau} s_n(t-\tau)v_n(t-\tau) - \delta v_n(t). \tag{A.1c}$$

When parasites have long latency periods, (A.1a-c) can be solved by splitting parasites by their generation where the first generation of parasites in season n, $v_{1,n}$ produce the second generation of parasites in the season, $v_{2,n}$. The number of $v_{2,n}$ parasites remaining at the end of the season will give rise to next season's initial parasite population. The dynamics for this case are given by

$$\frac{ds_n}{dt} = \hat{s}(n)g(t_0, t_l) - \mu_s s_n(t) - \alpha s_n(t)v_{1,n}(t) - l s_n(t), \tag{A.2a}$$

$$\frac{da_n}{dt} = l s_n(t) - \mu_a a_n(t), \tag{A.2b}$$

$$\frac{dv_{1,n}}{dt} = -\delta v_{1,n}(t). \tag{A.2c}$$

$$\frac{dv_{2,n}}{dt} = \alpha\beta e^{-\mu_s \tau} s_n(t-\tau)v_{1,n}(t-\tau) - \delta v_{2,n}(t). \tag{A.2d}$$

We make the common assumption for free-living parasites that the removal of parasites through transmission (α) is negligible(Anderson and May 1981; Dwyer 1994; Caraco and Wang 2008), *i.e.* (A.2c) ignores the term $-\alpha s(t)v_{1,n}(t)$.

(A.2a-d) is solved analytically by describing host emergence using a uniform distribution

$$g(t_0, t_l) = \begin{cases} \frac{1}{t_l} & t_0 \le t \le t_0 + t_l \\ 0 & t_0 + t_l < t \end{cases} \tag{A.3}$$

To solve the dynamics during the host's activity period, we first find the analytical solution for $v_{1,n}(t)$:

$$v_{1,n}(t) = \hat{v}e^{-\delta t}$$

We use $v_{1,n}(t)$ to find the time-dependent solution for $s_n(t)$ and $a_n(t)$. We can then plug the time-dependent solution for $s_n(t)$ to find the time-dependent solution for $v_{2,n}(t)$. Only parasites that infect hosts from $t_0 < t < T - t_0 - \tau$ have enough time to release progeny before the end of the season. For $\tau < T - t_0 - t_l$, parasites that infect hosts during host emergence ($t_0 < t \leq t_0 + t_l$) have time to release progeny before the end of the season as well as some parasites who infect hosts after host emergence has ended ($t > t_0 + t_l$). For $\tau > T - t_0 - t_l$, only some parasites that infect hosts during host emergence ($t_0 < t \leq t_0 + t_l$) have time to release progeny before the end of the season. Thus, two separate solutions are required depending on whether τ is greater or less than $T - t_0 - t_l$. We first consider the case where $\tau < T - t_0 - t_l$:

$$s_n(t) = \begin{cases} \frac{\hat{s}}{t_l} e^{(-\mu_s(t-t_0) + \frac{\alpha \hat{v} e^{-\delta t}}{\delta})} \int_0^{t-t_0} e^{(\mu_s u - \frac{\alpha \hat{v} e^{-\delta u}}{\delta})} du & t_0 < t < t_0 + t_l \\ s_n(t_0 + t_l) e^{(-\mu_s(t - t_0 - tl) - \frac{\alpha \hat{v} e^{-\delta(t + t_0 + t_l)}(-1 + e^{\delta t})}{\delta})} & t_0 + t_l \leq t < T \end{cases}$$

$$a_n(t) = \begin{cases} \frac{\hat{s} l}{t_l} e^{-\mu_a(t-t_0)} \int_0^{t-t_0} e^{-(\mu_s - \mu_a + l)u + \frac{\alpha \hat{v} e^{-\delta u}}{\delta}} \int_0^u e^{(\mu_s + l)x - \frac{\alpha \hat{v} e^{-\delta x}}{\delta}} dx du & t_0 < t < t_0 + t_l \\ \frac{\hat{s} l}{t_l} e^{-\mu_a(t-t_0-t_l)} \int_0^{t-t_0-t_l} e^{-(\mu_s - \mu_a + l)u + \frac{\alpha \hat{v} e^{-\delta u}}{\delta}} \int_0^u e^{(\mu_s + l)x - \frac{\alpha \hat{v} e^{-\delta x}}{\delta}} dx du & t_0 + t_l \leq t < T \end{cases}$$

$$v_{2,n}(t) = \begin{cases} \frac{\alpha \beta e^{-\mu_s \tau} \hat{v} \hat{s}}{t_l} e^{-\delta(t - t_0 - \tau)} \int_0^{t-t_0-\tau} e^{(-\mu_s u + \frac{\alpha \hat{v} e^{-\delta u}}{\delta})} \int_0^u e^{(\mu_s x - \frac{\alpha \hat{v} e^{-\delta x}}{\delta})} dx du & t_0 + \tau < t < t_0 + t_l \\ e^{-\delta(t - t_0 - t_l - \tau)} (v_{2,n}(t_0 + t_l) + \alpha \beta e^{-\mu_s \tau} \hat{v} s_n(t_0 + t_l) \int_0^{t - t_0 - t_l - \tau} e^{-\frac{\alpha \hat{v} e^{-\delta(u + t_0 + t_l)}(-1 + e^{\delta u})}{\delta} - \delta t_l - \mu_s u} du) & t_0 + t_l \leq t < T \end{cases}$$

where $s_n(t_0 + t_l)$ and $v_{2,n}(t_0 + t_l)$ are the densities of s_n and $v_{2,n}$ when the emergence period of s_n ends.

For $\tau > T - t_0 - t_l$, only some of the parasites that infect hosts from $t_0 < t < t_0 + t_l$ have enough time to release progeny before the end of the season. $v_{2,n}(t)$ are thus only produced from infections that occurred from $t_0 < t < t_0 + t_l$. The solution for $v_{2,n}(t)$ in this case is

$$v_{2,n}(t) = \frac{\alpha \beta e^{-\mu_s \tau} \hat{v} \hat{s}}{t_l} e^{-\delta(t - t_0 - \tau)} \int_0^{t - t_0 - \tau} e^{(-\mu_s u + \frac{\alpha \hat{v} e^{-\delta u}}{\delta})} \int_0^u e^{(\mu_s x - \frac{\alpha \hat{v} e^{-\delta x}}{\delta})} dx du \qquad t_0 + \tau < t < T$$

When $\tau < T - t_0 - t_l$, the parasite density at the end of the season is given by

$$v_{2,n}(T) = e^{-\delta(T - t_0 - t_l - \tau)} (v_{2,n}(t_0 + t_l) + \alpha \beta e^{-\mu_s \tau} \hat{v} s_n(t_0 + t_l) \int_0^{T - t_0 - t_l - \tau} e^{-\frac{\alpha \hat{v} e^{-\delta(u + t_l)}(-1 + e^{\delta u})}{\delta} - \delta t_l - \mu_s u} du)$$

When $\tau > T - t_0 - t_l$, the parasite density at the end of the season is given by

$$v_{2,n}(T) = \frac{\alpha \beta e^{-\mu_s \tau} \hat{v} \hat{s}}{t_l} e^{-\delta(T - t_0 - \tau)} \int_0^{T - t_0 - \tau} e^{(-\mu_s u + \frac{\alpha \hat{v} e^{-\delta u}}{\delta})} \int_0^u e^{(\mu_s x - \frac{\alpha \hat{v} e^{-\delta x}}{\delta})} dx du$$

End of season host density ($a(T)$) is given by

$$a_n(T) = \frac{\hat{s}l}{t_l} e^{-\mu_a(T-t_0-t_l)} \int_0^{T-t_0-t_l} e^{-(\mu_s+l)u+\frac{\alpha\hat{v}e^{-\delta u}}{\delta}} \int_0^{u} e^{(\mu_s+l)x-\frac{\alpha\hat{v}e^{-\delta x}}{\delta}} dx du$$

The total population of uninfected hosts who have matured to the second life stage by the end of the season, $a_n(T)$, reproduce and give rise to next season's host cohort ($\hat{s}$), given by the map

$$\hat{s}(n) = \frac{\epsilon\sigma a_{n-1}(T)}{1+\rho a_{n-1}(T)} \tag{A.4}$$

where ϵ is the probability that hosts survive to the next season, σ is host fecundity and ρ is the density dependent parameter.

Appendix B

In Appendix B we find analytical solutions for equations (2a-e) from the main text to study the evolution of host phenological traits. Note that we primarily used numerical simulations in the main text to determine the outcome of host evolution as this analytical solution only holds when the parasite has long enough τ that it is only able to complete one round of infection per season.

$$\frac{ds_n}{dt} = \hat{s}(n)g(t_0,t_l) - \mu_s s_n(t) - \alpha s_n(t)v_n(t) - ls_n(t), \tag{B.1a}$$
$$\frac{da_n}{dt} = ls_n(t) - \mu_a a_n(t), \tag{B.1b}$$
$$\frac{ds_{n,m}}{dt} = \hat{s}_{n,m}(n)g(t_{0m},t_{lm}) - \mu_s s_{n,m}(t) - \alpha s_{n,m}(t)v(t) - ls_{n,m}(t), \tag{B.1c}$$
$$\frac{da_{n,m}}{dt} = ls_{n,m}(t) - \mu_a a_{n,m}(t), \tag{B.1d}$$
$$\frac{dv_n}{dt} = \alpha\beta e^{-\mu_s\tau}v_n(t-\tau)(s_n(t-\tau)+s_{n,m}(t-\tau)) - \delta v_n(t). \tag{B.1e}$$

with initial conditions: $s_n(0)=0, a_n(0)=0, s_{m,n}(0)=0, a_{m,n}(0)=0, v_{1,n}(0^+)=v_{2,n}(0^-), v_{2,n}(\tau)=0$. m subscripts refer to the invading mutant host and its corresponding traits.

We determine the invasion fitness of a rare mutant host depending on its density at the end of the season it

was introduced. Mutant density is found by solving (B.1c, B.1d and B.1e)

$$v_{1,n}(t) = v_{1,n}(0)e^{-\delta t} \qquad\qquad 0 < t < T$$

$$s_{m,n}(t) = \begin{cases} \frac{\hat{s}}{t_{lm}} e^{(-\mu_s(t-t_{0m})+\frac{\alpha\hat{v}e^{-\delta t}}{\delta})} \int_0^{t-t_{0m}} e^{(\mu_s u - \frac{\alpha\hat{v}e^{-\delta u}}{\delta})} du & t_{0m} < t < t_{0m} + t_{lm} \\ s_{m,n}(t_{0m}+t_{lm}) e^{(-\mu_s(t-t_{0m}-tlm)-\frac{\alpha\hat{v}e^{-\delta(t+t_{0m}+t_{lm})}(-1+e^{\delta t})}{\delta})} & t_{0m}+t_{lm} \le t < T \end{cases}$$

$$a_{m,n}(t) = \begin{cases} \frac{\hat{s}l}{t_{lm}} e^{-\mu_a(t-t_{0m})} \int_0^{t-t_{0m}} e^{-(\mu_s-\mu_a+l)u+\frac{\alpha\hat{v}e^{-\delta u}}{\delta}} \int_0^u e^{(\mu_s+l)x-\frac{\alpha\hat{v}e^{-\delta x}}{\delta}} dx du & t_{0m} < t < t_{0m}+t_{lm} \\ \frac{\hat{s}l}{t_{lm}} e^{-\mu_a(t-t_{0m}-t_{lm})} \int_0^{t-t_{0m}-t_{lm}} e^{-(\mu_s-\mu_a+l)u+\frac{\alpha\hat{v}e^{-\delta u}}{\delta}} \int_0^u e^{(\mu_s+l)x-\frac{\alpha\hat{v}e^{-\delta x}}{\delta}} dx du & t_{0m}+t_{lm} \le t < T \end{cases}$$

where $s_{m,n}(t_{0m}+t_{lm})$ is the density of $s_{m,n}$ when its emergence period of ends at $t = t_{0m}+t_{lm}$.

The invasion fitness of a rare mutant host is given by the density of $a_{m,n}$ produced by the end of the season. The mutant host invades if the density of $a_{m,n}$ produced by time T is greater than or equal to the initial $s_{m,n}(0) = 1$ introduced at the start of the season ($a_{m,n}(T) \ge 1$), following

$$a_{m,n}(T) = \frac{\hat{s}l}{t_{lm}} e^{-\mu_a(T-t_{0m}-t_{lm})} \int_0^{T-t_{0m}-t_{lm}} e^{-(\mu_s-\mu_a+l)u+\frac{\alpha\hat{v}e^{-\delta u}}{\delta}} \int_0^u e^{(\mu_s+l)x-\frac{\alpha\hat{v}e^{-\delta x}}{\delta}} dx du$$

Appendix C

In Appendix C we describe the numerical methods used to find evolutionary attractors. We follow the same approach as previous work (MacDonald et al. 2022; MacDonald and Brisson 2022a; **?**) and define mutant invasion fitness as the density of the emerging mutant host ($s_{m,n}$) in season $n+1$ ($\hat{s}_m(n+1)$) produced by a single invading mutant host ($\hat{s}_m(n+1) = s_{m,n}(0) = 1$) introduced in season n. That is, mutant invasion fitness is the number of susceptible mutant hosts $\hat{s}_m(n+1)$ that were produced in season n by $a_{m,n}(T)$ in the environment set by the resident host at equilibrium density $\hat{s}^*$. Thus, a susceptible mutant host ($s_{m,n}$) introduced in season n only invades if $\hat{s}_m(n+1)$ is greater than or equal to the initial $s_{m,n}(0) = 1$ introduced at the start of the season n ($\hat{s}_m(n+1) \geq 1$).

It is not possible to derive an algebraic expression for mutant invasion fitness in this study as it was in previous studies (MacDonald et al. 2022). To determine evolutionary attractors for host phenology traits (t_0, t_l) we instead numerically find the density of $\hat{s}_m(n+1)$ produced after one season in an environment set by the resident host at equilibrium density (s^*). As in the previous analytical approach (MacDonald et al. 2022; MacDonald and Brisson 2022a; **?**), $\hat{s}_m(n+1) = 1$ produced by a single mutant introduced in season n corresponds to a neutral mutant, $\hat{s}_m(n+1) > 1$ corresponds to a mutant-resident pair in which the mutant parasite can invade and replace the resident, $\hat{s}_m(n+1) < 1$ corresponds to a mutant-resident pair that drives the mutant parasite extinct. Values of t_0 and t_l corresponding to attractors prevent small effect mutants with higher and lower traits from invading. For example, when evolutionary stable attractors occur where resident t_0 is t_{0r} and mutants with $t_{0m} = t_{0r} + 0.01$ and $t_{0m} = t_{0r} - 0.01$ cannot invade.

This method has been used previously in scenarios that generate multiple evolutionary attractors (**?**) by identifying all trait values that prevent small-effect mutants with higher and lower values from invading. We did not find evidence that multiple attractors or repellors exist in this study (*i.e.* no evidence of evolutionary bistability or evolutionary branching) (Metz et al. 1992; Geritz et al. 1998). In addition, we verified this finding by inspecting pairwise invasibility plots which only ever have one evolutionary attractor.

Appendix D

In Appendix D we present plots that show the robustness of optimal host emergence starts and optimal host emergence period lengths to changes in three key parameters: parasite transmission rate (α), host fecundity (σ) and the density dependent parameter (ρ) that maps host densities from one season to the next.

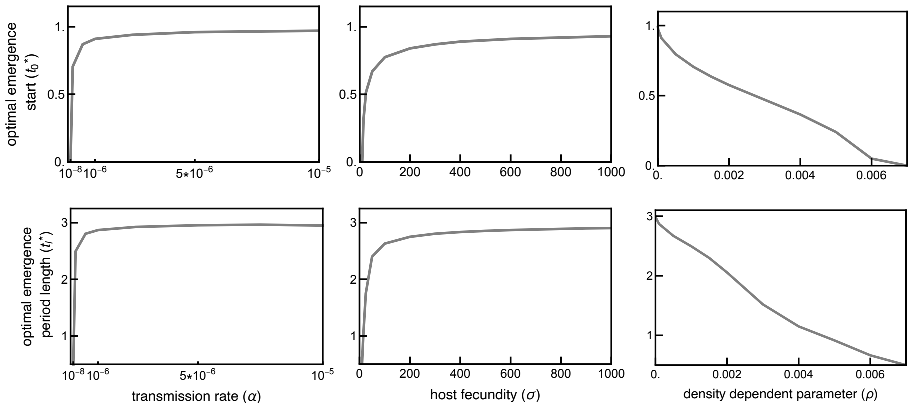

Figure 6: Extreme parameter values decrease the impact of parasites on host phenology evolution. Changes in the parasite transmission rate has minimal impact on optimal host emergence start (t_0) and emergence period length (t_l), except at extremely small parameter values ($\alpha < 10^-7$). Small α decreases parasite density as parasites do not infect hosts as readily; thus only small shifts in host phenology are necessary to decrease infection risk. Changes in host fecundity (σ) also have minimal impact on optimal t_0 and t_l, except at extremely small parameter values ($\sigma < 100$). Small σ decreases host density and thus parasite infections (as transmission is density dependent). Small host densities driven from small σ make small shifts in host phenology sufficient to reduce parasite infection risk. Changes in the between-season host density dependent parameter (ρ) have a large impact on optimal t_0 and t_l. Smaller shifts in host phenology are optimal as the strength of ρ increases. Large ρ decreases host density, similar to small $sigma$, thus making small shifts are sufficient to decrease parasite infection risk. Black line: $\tau = 1.5$, gray line: $\tau = 3$, $t_0 = 0, t_l = 0.5$ (unless varying), $n = 300$, all other parameters are the same as in Table 1.
